# Analytical Assessment of Bioelements in Various Types of Black Teas from Different Geographical Origins in View of Chemometric Approach

**DOI:** 10.3390/molecules26196017

**Published:** 2021-10-04

**Authors:** Wojciech Koch, Wirginia Kukula-Koch, Marcin Czop, Tomasz Baj, Janusz Kocki, Piotr Bawiec, Roser Olives Casasnovas, Anna Głowniak-Lipa, Kazimierz Głowniak

**Affiliations:** 1 Department of Food and Nutrition, Medical University of Lublin, 4a Chodźki Str., 20-093 Lublin, Poland; piotr.bawiec@wp.pl (P.B.); roserolives@gmail.com (R.O.C.); 2 Department of Pharmacognosy with Garden of Medicinal Plants, Medical University of Lublin, 1 Chodźki Str., 20-093 Lublin, Poland; virginia.kukula@gmail.com (W.K.-K.); tomasz.baj@umlub.pl (T.B.); 3 Department of Clinical Genetics, Medical University of Lublin, 11 Radziwiłłowska Str., 20-093 Lublin, Poland; marcin.czop@umlub.pl (M.C.); janusz.kocki@umlub.pl (J.K.); 4 Faculty of Pharmacy and Food Science, University of Barcelona, Joan XXIII, 27-31, 08028 Barcelona, Spain; 5 Department of Cosmetology, University of Information Technology and Management in Rzeszów, Kielnarowa 386a, 36-020 Tyczyn, Poland; aglowniak@wsiz.edu.pl (A.G.-L.); kglowniak@pharmacognosy.org (K.G.)

**Keywords:** *Camellia sinensis*, theaceae, black tea, elements, PCA, FAAS

## Abstract

A comprehensive approach to the mineral composition of black teas of different origins was studied using the Flame Atomic Absorption Spectrometry (FAAS) method, supported by chemometric tools including Principal Component Analysis PCA) and Classification and Regression Trees (CART). Significant differences between the teas from seven countries (Japan, Nepal, Kenya, Iran, Sri Lanka, India, and China) were shown. K was the main element determined in all teas, with an average concentration of 11,649 mg/kg, followed by Ca, Mg and Mn. In general, regarding all investigated black teas, the element content was ranked in the following order: K > Ca > Mg > Mn > Fe > Na > Zn > Cu. The applied chemometric methods allowed us to recognize black tea clusters based on their mineral composition and place of cultivation, and allowed us to find correlations between particular elements in black teas. The performed analyses revealed interesting correlations between the concentration of various elements in black teas: K was negatively correlated with Na, Fe, Mn and Cu; K was positively correlated with the content of Ca and Mg. Significant positive correlations between Mn and Fe and Mn and Zn in the studied black tea samples were also revealed. It was shown that mineral composition may be a significant factor regarding the origin of the black tea, not only considering the country, but also the region or province.

## 1. Introduction

The tea plant *Camellia sinensis* L. (Kuntze) is a shrub widely cultivated in more than 40 countries across the world, however it prefers a warm, wet climate and a slightly acidic soil [1]. Depending on the production process, tea can be divided into seven major types: black, green, yellow, white, oolong, aged Pu-erh, and ripened Pu-erh [2]. Although there are over 1500 different varieties of tea types that differ from one another in taste and color, black tea accounts for approximately 75% of all tea production in the world, and therefore can be considered to be the most important type of tea [3,4,5,6]. The second most widespread tea type (with 20% global consumption, especially in Far East countries) is green tea that is produced from freshly harvested and non-fermented leaves [7]. Pu-erh, on the other hand, is considered one of the most aromatic types, having a spicy taste and distinct smell [8], which is especially popular in the Upper MeKong River Region of Yunnan Province (China), Laos, Vietnam, Myanmar, and India [9,10].

Black tea contains a large variety of active compounds, including organic and mineral components. The former group consists of simple polyphenols, called flavan-3-ols, or catechins, such as (–)-epicatechin gallate (ECG), (–)-epigallocatechin (EGC), or (–)-epigallocatechin-3-gallate (EGCG). During the fermentation process, condensed polyphenols that include theaflavins (TFs), thearubigins (TRs), and theabrownins (TBs) are formed. The latter compounds are responsible for the specific taste, color, and aroma of black tea infusions [11,12]. Methylxanthines, ex. Caffeine, or theophylline are also important organic constituents of the tea leaf, responsible for its analeptic and diuretic properties. The mineral composition of black tea is complex and includes bio-elements (Ca, Mg, K, Mn, Zn or Fe) and toxic elements (Pb, Cd or Ni). Their content is mostly dependent on the region of cultivation, usage of fertilizers, and pH of soil [13,14]. Thanks to the presence of various active biological compounds, black tea has numerous beneficial effects on health, including antiviral, antibacterial, anticancer, and protective properties against a variety of civilization diseases, including cardiovascular disorders and memory impairment [4,5,15,16,17].

Chemometric methods are widely used to classify, evaluate, and distinguish various samples, including natural products. Such a tool may be very helpful to fully characterize obtained spectrometric or chromatographic results and to reveal significant correlations and similarities. Among them, Principal Component Analysis (PCA), Classification and Regression Trees (CART), Cluster Analysis (CA), and Linear Discriminant Analysis (LDA) are the most frequently used [6,8,11].

Considering a wide diversity of black teas regarding their origin and chemical composition, the main purpose of this study was to assess the level of mineral bioelements (macro- and trace elements) in various original black teas using Flame Atomic Absorption Spectrometry (FAAS) and to find the correlations between the mineral composition of tea samples from different origins using the chemometric tools PCA and CART. The herein-presented results complement the recently published studies of authors that have focused on the differentiation of black teas based on their catechins’ composition [6] and green teas based on their organic and mineral content [18]. To the best of our knowledge, the present study is the first one showing the results of chemometric analysis in the search for correlations between the mineral composition and the origin of black teas, including the province or region of origin.

## 2. Results

The concentration of four macroelements (Na, K, Ca and Mg) and four trace elements (Cu, Zn, Mn and Fe) is presented in Table 1.

Among all of the investigated elements, K was determined to have the highest concentration, while Cu had the lowest. The investigated black tea samples also contained high amounts of Ca and Mg. In the case of trace elements, Mn was the element determined to have the highest quantity. On the other hand, Na was present in a very low concentration in all tea samples. In general, regarding all investigated black teas, the element content can be ranked in the following order: K > Ca > Mg > Mn > Fe > Na > Zn > Cu.

**Figure 1:**
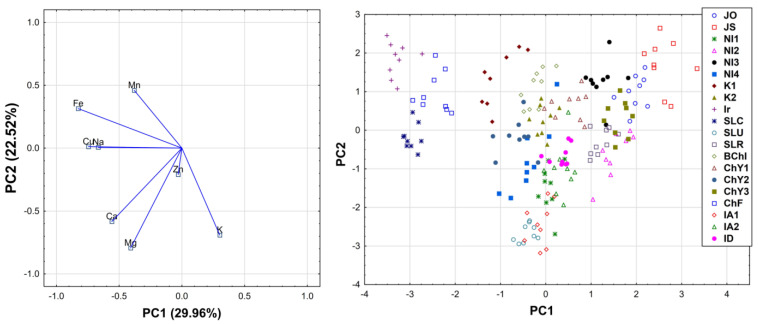
Loadings (on the **left**) and scores (on the **right**) of first two components of principal component analysis (PCA).

**Figure 2:**
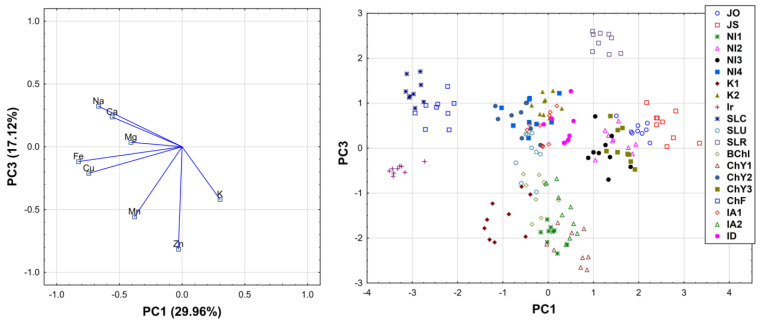
Loadings (on the **left**) and scores (on the **right**) of first and third components of principal component analysis (PCA).

A matrix consisting of rows that included the types of tea and columns that represented the elements was prepared from the obtained results. Based on the gathered data, the PCA analysis was performed, and explained 69.61% of data variability in the three principal components. PC1 explained 29.96% of the variation, PC2 22.52% of the variation, and PC3 17.12% of the variation (their equivalents were 2.40, 1.80, and 1.37, respectively (Figure 1 and Figure 2).

The first ingredient (PC1) separated two groups of teas. The first group represented tea samples of the type Ir, ChF, and SLC, as the samples containing high contents of Fe, Cu, Na, and Mn. The second group included JO, JS and NI3 teas, which contained a small amount of the elements, i.e., Ca, Mg, Fe, and Cu (a similar group was obtained by analyzing the second component (PC2)).

The second ingredient (PC2) separated a group of teas containing a marked quantity of Ca, Mg, and K, and this group included the tea samples of the SLU, IA1, IA2, NI1, NI4, ID type (Figure 1 and Figure 2).

The third component of PC3 also separated two groups of teas. The first group contained teas of the type NI1, BChI, K1, IA2, and ChY1, which were characterized by the presence of high amounts of Zn but low amounts of CCa and Na. The second group was represented by only one type of tea (SLR), which was low in Fe, Mn, Zn, and Cu (Figure 1 and Figure 2).

When analyzing the relationship between the number of investigated elements based on the correlation of factors, the following groups of correlating elements can be distinguished. The first group of samples gathered Na, Fe, and Cu, the amounts of which, in the tested types of tea, were positively correlated with one another. The next group included K-containing samples, the amounts of which were negatively correlated with the content of Na, Fe, Mn, and Cu (based on PC1). The following group contained Ca, Mg, and K, the amounts of which, in the tested types of tea, were positively correlated with one another. Additionally, the third group consisted of Fe and Mn-containing tea digests, the amounts of which, in the tested types of tea, were positively correlated with one another, but not correlated with the second group of samples (based on PC2). The next distinguished group was formed based on the content of Mn and Zn, the amounts of which, in the tested types of tea, were positively correlated with one another. An additional last group formed tea samples rich in Ca and Na, the amounts of which were positively correlated with each other, but were not correlated with the fourth group (based on PC3).

**Figure 3:**
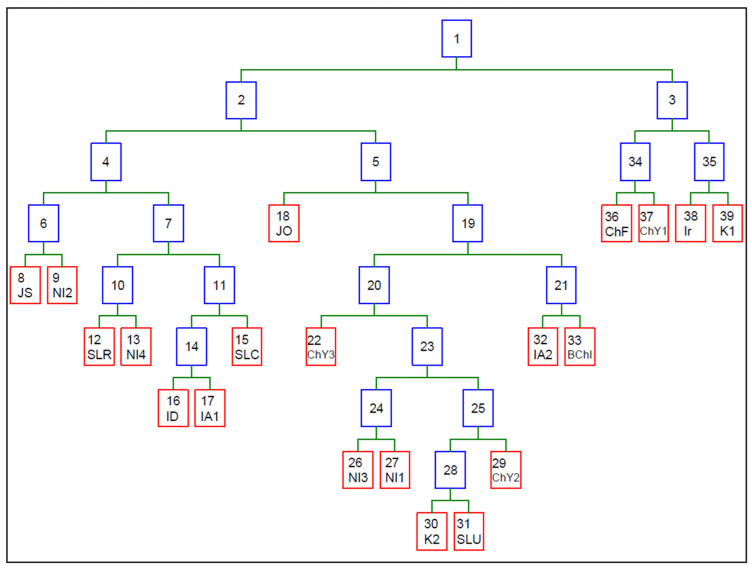
Classification tree for samples of tea. The number is the node number. (Figure S1 in the Supplementary Materials presents full version of the tree).

In the next stage of the data exploration of the content of macro- and trace elements in teas from different regions of the world, a non-parametric CART (Classification and Regression Trees) analysis was performed (Figure 3). The distribution attributes were the content of the analyzed elements, determined using the FAAS method. The contents of the analyzed elements in 180 samples constituted the root of the binary tree. As a result of constructing the tree, the number of splits (=19) and number of terminal nodes (=20) were obtained. The variable predictors in the tree structure were (in order of importance): Ca > Fe > Na > Zn > Mn > Cu > K > Mg. A detailed analysis of the split attributes for these samples is presented in Table 2.

The exploration of discriminatory data showed a high convergence between tea samples from India (ID and IA1), Nepal (NI3 and NI1), and China (CHF and CHY1) for the selected attributes.

## 3. Discussion

### 3.1. Macro- and Trace Element Content in Black Teas

The previous study of the authors, which focused on the elemental content of green teas [18], revealed the following order of metals’ concentration: K > Mg > Ca > Mn > Fe > Na > Zn > Cu. Surprisingly, the obtained data for green tea samples resembled the herein described results. The order of the elements’ concentration was as expected, if we consider their functions from the point of view of plant physiology. K is required in large amounts for plant growth. K-deficient plants showed various metabolic disorders, including a strong increase in soluble sugar concentration, an accumulation of several basic or neutral amino acids, and increased total amino acid and protein content. Moreover, a significant reduction in pyruvate and other organic acid concentrations was observed in the roots [19,20]. Mg plays a crucial role in plant chlorophyll biosynthesis and carbon fixation as a cofactor of a series of enzymes involved in carbon metabolism; thus, its concentration in plant tissues is predominantly high [21]. However, it should be noted that the balance between the concentration of the individual elements in the plant is very delicate, and that too high a concentration of one may reduce the content of another; for example, too high a concentration of K and Ca in the soil may result in a Mg deficiency [22]. In plants, Mn is one of seventeen essential elements for growth and reproduction, however its homeostasis is poorly known [23]. Tea leaves are widely known to contain high amounts of Mn; however, this element has very little mobility within the plant organism, and its remobilization from older to Mn-deficient young leaves is very difficult [24]. Fe is required for many biological functions in plant cells, including the synthesis of chlorophyll, and, as a cofactor of cytochromes, is engaged in electron transport. Although Fe concentration in well-aerated soils is predominantly high, it primarily forms highly insoluble ferric compounds at neutral pH levels and thus its bioavailability for plants is usually low, which may result in Fe chlorosis [25]. It should also be noted that micronutrients, such as predominantly Mn, Cu, Fe, and Zn, play a substantial role in plant immune defense [26,27,28]. Among them, Zn is considered the most important component, not only for plants, but also animal immune responses [29,30,31]. Mg and Ca were previously determined to be a very similar level to each other [18], which is in agreement with the results of the present research. This is understandable if we consider the technology of black tea production. Its main stage includes the fermentation process, during which the majority of simple catechins are degraded to polymers, such as theaflavins, thearubigins, and theabrownins, while the concentration of elements stays at the same level [12,32,33,34]. K was the element determined in the highest quantity in all investigated black tea samples, which is in agreement with other reports, suggesting that this is a major metal in both green and black tea [35,36]. In the present study, the mean concentration of K was very diverse, and was within the range of 8344–15,323 mg/kg. The lowest content was determined in the tea samples cultivated in Iran, while the highest in those that originated from Nepal (NI1). Black tea cultivated in the Uva region of Sri Lanka also contained high amounts of this element. A significant correlation between K content and the place of origin can be found, not only in the case of particular countries, but even specific regions (e.g., similar results for the Yunnan province and Assam region).

From all macroelements, Na was determined to be in the lowest concentration by far, however its concentration was very diverse, and the content in individual samples differed even 10 times, from 11.2 in CHY3 to almost 114 mg/kg in samples from the Fuijan province in China (CHF). Na concentration revealed in the present research was very similar to the one reported for green tea samples [18], and resembled the data reported by Brzezicha-Cirocka et al., who investigated 48 different black teas and determined the average level of Na at 83.2 mg/kg, which was much lower compared to Pu-erh or fruit teas [8]. In contrast to K, the level of Na was not correlated with the place of origin, and its content varied significantly, even between samples from the same region (e.g., teas from Ilam region in Nepal or Yunnan province in China). It is also interesting that the calculated K to Na ratio was very high, and, on average (in all teas), was 273:1, which was much higher compared to the same parameters for green teas (207:1) [18]. This indicates that both green and black tea may have a very positive impact on human health in terms of high K, low Na content, as was previously proved for other natural products, including ecological or organic cultivars [37].

The beneficial properties of black teas, especially towards cardiovascular diseases, may be even stronger if we consider the high Mg content in those natural products. The present study has revealed an average concentration of Mg at almost 1 g/kg, ranging from 822 in teas from Japanese Prefecture Shizuoka (JS) to 1233 mg/kg in samples from the Assam region in India. Moreover, teas from Iran and Sri Lanka (all regions) contained Mg at the level ≥1 g. The results obtained in the present research were similar to those obtained by Konieczyński et al. (1060–1600 mg/kg) for green teas, but much lower compared to black teas (1370–2140 mg/kg) [38]. Chu et al. also reported higher Mg content in green teas in the range of 1200–3000 mg/kg [35]. According to Pohl et al., the concentration of Mg in herbal teas was also higher, ranging from 1320 to 2280 mg/kg [39]. In general, the content of Mg differed significantly between all investigated black tea samples, and thus the content of this element may be considered to be one of the factors that differentiate the origin of the tea. This is a new finding, which is not in agreement with the conclusions stated by other authors, who did not differentiate black teas based on the content of Mg [38].

Ca was the most abundant element after K and Mg; its average concentration (including all black teas evaluated in the study) was 1272 mg/kg, within the range from 503.3 to 1856 mg/kg. Japanese black teas contained by far the lowest concentration of this metal, whereas samples from all regions of Sri Lanka and the Assam region in India were characterized by a high concentration of Ca. Although the concentration of this macroelement was correlated with the origin and region of cultivation, significant differences between the samples from the same region were also noticed—three products from Ilam in Nepal were characterized by a moderate Ca concentration (NI1, NI2, and NI3), whereas NI4 samples contained much higher amounts of this element (1532 mg/kg). Similar conclusions can be drawn by assessing the Ca content of samples from the Yunnan region in China. Ca concentration determined in the present research was lower in comparison to green teas, in which the average concentration for various products was within the range of 817–2639 mg/kg [18]. Brzezicha-Cirocka et al. revealed higher concentrations of Ca in black teas containing fruits, as well in Pu-erh and fruit teas, however the concentration of this element was very diverse, and was within the range of 1200–14,370 mg/kg [8]. According to Dambiec et al., the level of Ca in various black teas from a Polish market ranged from 1700 to 2820 mg/kg. This study involved mainly blended teas of indefinite origin, however the highest concentration of Ca was revealed for black tea, for which the origin was established as South India [40]. In another study performed by Brzezicha-Cirocka et al., the level of Ca in original black teas was 1550–1610 mg/kg in original teas, and no statistical differences between products from different origins were revealed. Interestingly, the average level of Ca in blended teas was much higher in comparison to original products (2640 mg/kg) [41]. The results of the present study, as well as data obtained by other authors, indicate that the concentration of Ca in black teas is very differentiated, and connected not only to origin, but also to the type of product and manufacturer.

Regarding the content of trace elements in the studied black teas, Mn was by far the element characterized by the highest concentration, which is well known and in agreement with many previous reports [8,18,40,41]. High content of Mn in black tea may support antioxidant activity of the body, as this metal is a crucial constituent of superoxide dismutase [6,18]. The mean concentration of this element in all black teas was calculated as 447.7 mg/kg, with the highest content measured for the samples from the Yunnan province in China (CHY1) (846.3 mg/kg) and the lowest for tea samples from Assam in India (IA1) (202.8 mg/kg). Moreover, black tea from Kenya (K1) contained high amounts of Mn (824.7 mg/kg). Teas with low or moderate content of Mn were originated from Japan, Nepal, and Sri Lanka. Brzezicha-Cirocka et al. also revealed significant differences in the concentration of Mn related to the place of origin, and also noticed a high concentration of this element in samples from Kenya [41]. A high quantity of Mn in tea leaves is very characteristic, and therefore all types of tea (black, green, or Pu-erh) are considered significant dietary sources of this element for the general population [8,18,41,42]. The presence of organic antinutritive substances such as oxalates or tannins in tea leaves may decrease the absorption of Mn, however studies performed by Powell et al. in simulated intestinal conditions proved that bioavailability of Mn from tea infusion is still high (40%), and therefore tea should be considered as the main dietary source of this element to humans [42].

Fe was the trace element determined in the highest concentration in all black teas after Mn, with an average content of above 70 mg/kg. However, its concentration varied in a very wide range from 26.9 to 179.5 mg/kg for Japanese and Iranian products, respectively. Fe content in Pu-erh teas (loose and bag forms), fruit teas, and black teas containing fruits [8] were close to the results of the present research. However, the obtained results were much higher in comparison to values obtained by other authors regarding Fe content in black teas, in which an average content was within the range of 4.3–9.0 mg/kg [41]. The latter study, however, did not find significant differences between Fe content in samples from various origins. The present research confirmed that Fe content in black teas varies significantly, considering not only the country of origin, but also regions and plantations. Moreover, the results of the present determinations proved that tea leaves might be rich in Mn and Fe, although previous studies have suggested that high Fe levels can cause Mn deficiency in tea plants [41,43].

Black tea samples from Nepal (NI1) contained the highest amounts of Zn, whereas the samples from Sri Lanka were characterized by the lowest level of this trace element. In general, the concentration of Zn in various products was rather similar, and was not that differentiated, as in the case of Fe. The selected products from the Ilam region in Nepal, the Yunnan province in China, and the Assam region in India contained higher amounts of Zn. What is interesting, blended tea was also characterized by a high concentration of Zn. The obtained results were similar to the values presented in other reports, which also revealed the highest content of Zn in teas from China and India, and also in blended products [41,44]. This may indicate that processing observed during the production process may significantly increase the content of various trace metals, including Zn.

Furthermore, the content of Cu was very differentiated in the studied samples. The lowest content of this element was revealed in the Japanese tea (JO) and the highest in Iranian samples. Concentration of Cu was strictly correlated with the place of origin, however significant differences were also revealed between samples from the same country and region (e.g., teas from Kenya, Sri Lanka, or India). Similar to other elements, significant amounts of Cu were also determined in a blended product, which may suggest contamination during the production process. The obtained results were close to other reports, indicating high amounts of Cu in teas from Sri Lanka, Kenya, and India [41,45].

The levels of trace metals revealed in the present study were on a similar level in comparison to green teas [18], and thus the thesis raised by some authors that the latter teas, as non-fermented products, are characterized by a lower concentration of trace elements in comparison to black teas [46] was not confirmed. On the other hand, it was proved that blended, commercial products, which have an unknown geographical origin, are very often contaminated with high levels of various trace metals, which is in agreement with the reports of other authors [41,46].

### 3.2. Chemometric Analysis of the Obtained Data

The performed PCA analysis, supported by ANOVA analysis, revealed significant and interesting relationships between the investigated teas, based on their elemental composition and origin. Teas cultivated in the Assam region (IA1 and IA2), as well as the Darjeeling region (ID) in India, based on the high concentrations of Ca, Mg, and K, were considered similar. What is interesting is that this group of teas also contained samples from the Ilam region in Nepal (NI1 and NI4) and tea from Sri Lanka (Uva region). Japanese teas (JO and JS) were significantly separated from other samples, as products containing low amounts of Ca, Mg, Fe, and Cu. Interestingly, teas from Iran, the Fuijan province in China, and Central Province in Sri Lanka, were also significantly separated from other samples, as products containing high amounts of Fe, Cu, Na, and Mn. Interestingly, one blended product which was used in the present study was revealed to contain a mixture of two teas which were similar to that from the Yunnan province in China and the Assam region in India, which is in agreement with the producent declaration that this product was produced using teas cultivated in these two specific countries. This indicates that the applied method may be also useful for the evaluation of blended teas.

The performed chemometric analysis also revealed interesting correlations between the concentrations of various elements in black teas. As a result, it was noted that K was negatively correlated with Na, Fe, Mn, and Cu. K, on the other hand, was positively correlated with the content of Ca and Mg. Significant positive correlations between Mn and Fe, and Mn and Zn, in the studied black tea samples were also revealed.

The present study shed new light on the application of chemometrics in the discrimination of the origin of black teas. The previously performed studies [41,45] showed that the determination of the mineral composition in combination with chemometric tools may be very helpful in evaluating the geographical origin of black teas. The herein-described analyses have revealed that not only country, but also province or region, has a noticeable impact on the composition of tea samples, as some of the products from the same country, but different regions, were considered significantly different, i.e., teas originating from the Fuijan and Yunnan provinces in China, or teas cultivated in various parts of Sri Lanka. However, it should be remembered that even the use of advanced statistical methods may not bring the expected results, as the mineral composition of tea is related not only to the cultivation area, but also to the plant variety and the fertilization conditions used, which significantly affects its mineral composition, and eventually distorts the results of statistical analysis. Present results confirm this assumption, as some of the samples from the Yunnan province in China or the Ilam region in Nepal, although coming from the same region, were considered different.

The currently used analytical methods allow the assessment of both qualitative and quantitative composition with high precision. However, with the development of research methods, the amount of data received increases. Chemometric analysis allows for the multidimensional evaluation of data. In addition to the typical methods for this type of analysis, such as principal component analysis (PCA); cluster analysis (CA); linear discriminant analysis (LDA); k-nearest neighbour (kNN); and artificial neural networks (ANN), the classification and regression trees (CARTs) method is also mentioned [47]. For example, Zhand et al. used both linear and nonlinear models for discriminatory purposes. The authors showed that the ANN, CART, and random forest (RF) models showed greater efficiency compared to the linear models in determining the multicomponent fingerprint for different apple cultivars. The authors used the content of 15 elements to construct the models [48]. Vanderschueren et al. also used the CART analysis, on the basis of which they classified the chocolate samples according to the continent of cocoa origin, taking into account the content of bioelements (Ba, Cd, Mo, Sr, and Zn). The decision tree was used to both classify unknown samples and to create a fingerprint in terms of the composition of elements correlated with the place of origin [49].

Macro- and trace elements may be suitable attributes of discriminant methods, however numerous test samples are needed. In this study, the CART analysis allowed to us determine the order of bioelements differentiating the studied teas.

## 4. Materials and Methods

### 4.1. Plant Material

Black teas evaluated in the study were purchased in Poland in 2018–2019 from professional tea shops specializing in the sale of original high-quality products, where the origin of tea is guaranteed (including region or plantation, where it is needed). The study involved nineteen original black teas, cultivated in seven countries (Japan, China, Sri Lanka, Iran, Nepal, India, and Kenya). On purpose, one blended product was used for comparison. In total, the composition of twenty various black teas was evaluated. A precise description of the studied material was presented in Table 2, including a voucher specimen number given to each tea. Except one blended product, no teas with unknown origin or cultivation area were used in the study. It should be emphasized that the aim of this study was not to compare between original and blended products. However, the latter was used to check whether their mineral composition significantly differs from the original ones, which may influence place of origin. For each product, three different batches were used, and, from each batch, three different samples were taken. All studies were made in triplicate. In total, 180 black tea samples were used in the study (20 products x 3 batch numbers purchased x 3 samples taken for the analysis). Characterization of products used in the study was presented in Table 3. 

### 4.2. Reagents

All reagents were of Suprapur Grade and were purchased from Merck (Darmstadt, Germany). The calibration curves of every investigated element were plotted using certified stock solutions (1000 mg/L Merck, Darmstadt, Germany). High purity deionized water (resistivity 18.2 MWcm), which was used during all spectroscopic analyses (including the digestion process) was obtained with the Merck Millipore Direct-Q 3 UV system (Merck Millipore, MA, USA). Polypropylene laboratory vessels were used to store the obtained digests.

### 4.3. Elemental Analysis

#### 4.3.1. Sample Preparation

Every tea batch was first carefully ground in a ceramic mortar. Next, three portions of 10 g each were weighted into quartz crucibles, dried in an electrical dryer at a temperature of 105 °C, and subjected to the digestion process.

#### 4.3.2. Digestion Process

The digestion process and the elemental determinations were performed according to the previously described protocols [18,19]. Briefly, the samples were ashed in the muffle furnace at 450 °C. The organic particles were later removed using the 30% solution of nitric (V) acid, which was then evaporated, and the samples were re-heated at 250 °C for the following 2 h. The obtained ash was dissolved in 10% hydrochloric acid, filtered, and diluted with high purity deionized water to the final volume of 25 mL.

#### 4.3.3. The Analytical Determinations Performed by Flame Atomic Absorption Spectrometry (FAAS)

The content of all investigated elements was investigated using FAAS method using SOLAAR M5 apparatus (Thermo Scientific, Waltham, MA, USA). For each element, the appropriate dilutions were used, if needed. In the case of Ca, a spectral buffer containing Lanthanum trichloride was used.

The method was previously validated and checked for its accuracy and precision for elemental determinations in tea samples, which was described in a previous paper [18].

#### 4.3.4. Statistical Analysis

The level of statistical significance was set at *p* ≤ 0.05. The statistical analysis was performed using Microsoft Excel 2010 and Statistica 13.3 (StatSoft, Krakow, Poland) programs. All results were presented as mean ± standard deviation (SD) and range. Data were analyzed for normal distribution using the Shapiro–Wilk test. The statistical significance of elements between types of teas were analyzed using one-way analysis of variance (ANOVA, with one qualitative factor) followed by Tukey’s test. To reduce the number of variables and to detect the structure of relationships between variables, principal component analysis (PCA) was used. The selection of the number of components was carried out in accordance with the Kaiser criterion and the scree plot.

Classification and Regression Trees (CART) was carried out using the Statistica 13.3 program (StatSoft, Krakow, Poland), and the Data Mining module was carried out using the Gini measure and estimated prior probabilities.

## 5. Conclusions

Applying the FAAS method enabled the determination of various elements in black teas with appropriate accuracy and precision. The obtained results revealed significant differences between the concentration of macro- and trace elements in various black teas of different origins. K was the element determined to be in the highest concentration, followed by Ca, Mg, and Mn. The applied chemometric tools (ANOVA, PCA, and CART) allowed for the recognizing of black teas clusters, based on their mineral composition and place of cultivation. It was revealed that mineral composition may be a significant factor regarding the origin of the black tea, not only considering the country, but even the specific region or province. However, the applied methodology also has some limitations, as the mineral composition is not only affected by the origin, but also by other factors, which may disturb the obtained results. The classification and regression tree (CART) method was proven to be an equally useful tool for data mining. This method can be complementary to parametric methods in discriminant analysis. The application of spectrometric determinations of elements in combination with chemometric methods may a useful and practical tool in the recognition and evaluation of black teas, including their place of cultivation.

## Figures and Tables

**Table 1:** The average content of macro- and trace elements in black teas from various origins. Each value represents mean ± SD and range. Means not sharing the same letter in a column are significantly different at *p* ≤ 0.05; Tukey post-hoc test.

Type of Tea	Macroelements [mg/kg]				Trace Elements [mg/kg]			
	Na	K	Ca	Mg	Cu	Zn	Mn	Fe
**JO**	49.11 ± 2.41 ^FG^	11,158.11 ± 1167.50 ^BCDEF^	842.56 ± 135.68 ^B^	877.12 ± 45.51 ^ABC^	5.30 ± 0.19 ^A^	24.42 ± 0.91 ^CD^	443.46 ± 16.24 ^F^	26.91 ± 3.51 ^A^
	46.10–52.80	10,133.00–13,142.00	711.90–1088.00	820.30–965.60	5.15–5.77	23.20–26.10	421.20–465.20	22.30–32.20
**JS**	18.74 ± 1.79 ^B^	10,577.44 ± 1888.93 ^BCD^	503.44 ± 70.52 ^A^	821.64 ± 76.10 ^A^	8.94 ± 0.74 ^B^	21.94 ± 1.34 ^BC^	302.52 ± 18.69 ^D^	32.92 ± 4.26 ^AB^
	16.20–21.20	8112.00–13,298.00	411.40–628.40	711.40–907.90	7.55–9.95	20.20–23.50	278.00–326.50	27.40–39.00
**NI1**	53.24 ± 3.70 ^G^	15,323.11 ± 1231.67 ^H^	1150.33 ± 73.42 ^CD^	1068.08 ± 107.31 ^FG^	12.46 ± 0.18 ^DE^	35.81 ± 1.61 ^G^	395.22 ± 29.98 ^E^	94.94 ± 12.56 ^E^
	48.90–59.50	13,991.00–17,878.00	1021.00–1226.00	922.60–1219.00	12.20–12.70	34.00–38.00	347.30–435.00	78.90–117.10
**NI2**	21.31 ± 1.54 ^BC^	12,195.33 ± 624.88 ^DEFG^	994.20 ± 139.99 ^BC^	1041.76 ± 108.42 ^EFG^	11.97 ± 0.38 ^DE^	25.73 ± 1.27 ^DE^	224.11 ± 14.70 ^AB^	36.08 ± 3.83 ^AB^
	18.90–23.20	11,259.00–13,258.00	812.40–1189.00	914.40–1218.00	11.30–12.40	24.20–27.80	201.20–244.60	30.70–41.30
**NI3**	27.42 ± 3.48 ^CD^	11,199.44 ± 1497.08 ^BCDEF^	838.77 ± 77.81 ^B^	842.08 ± 68.49 ^AB^	11.10 ± 0.47 ^CD^	25.28 ± 1.52 ^DE^	359.41 ± 15.17 ^E^	79.42 ± 5.77 ^D^
	23.30–33.60	9211.00–13,711.00	709.20–951.00	705.40–922.00	10.50–11.80	22.60–27.60	332.40–378.40	71.10–87.70
**NI4**	53.44 ± 5.17 ^G^	12,752.67 ± 1448.30 ^EFG^	1532.00 ± 132.332 ^FGH^	1022.39 ± 118.45 ^DEFG^	11.78 ± 0.43 ^DE^	22.42 ± 1.14 ^BCD^	301.14 ± 20.63 ^D^	103.86 ± 11.98 ^EF^
	47.60–62.30	10,125.00–14,587.00	1311.00–1668.00	818.40–1195.00	11.10–12.40	20.50–24.20	265.40–328.40	85.50–120.20
**K1**	30.14 ± 2.96 ^D^	12,192.78 ± 1232.96 ^DEFG^	857.28 ± 95.68 ^B^	996.16 ± 101.34 ^CDEFG^	22.08 ± 2.64 ^I^	23.38 ± 1.69 ^CD^	824.72 ± 25.08 ^I^	99.52 ± 13.81 ^EF^
	25.40–34.80	10,545.00–13,685.00	719.40–980.20	889.40–1158.00	17.30–26.20	20.70–25.20	780.20–865.30	74.80–111.90
**K2**	45.19 ± 3.90 ^EF^	10,969.11 ± 715.67 ^BCDE^	1556.11 ± 131.55 ^GH^	932.21 ± 47.25 ^ABCDE^	12.57 ± 0.63 ^DE^	20.31 ± 1.35 ^B^	459.44 ± 15.13 ^F^	64.39 ± 8.25 ^C^
	39.70–50.70	10,115.00–12,547.00	1327.00–1693.00	878.40–1022.00	11.80–13.40	18.40–22.40	428.60–478.90	56.20–80.30
**Ir**	41.20 ± 3.40 ^E^	8344.56 ± 451.28 ^A^	1417.33 ± 30.48 ^F G^	1017.43 ± 68.03 ^DEFG^	23.87 ± 0.94 ^J^	24.10 ± 0.63 ^CD^	730.14 ± 15.35 ^H^	179.53 ± 12.92 ^H^
	36.10–45.80	7719.00–9211.00	1368.00–1455.00	932.80–1123.00	22.10–25.10	23.10–25.30	707.40–752.30	160.20–194.60
**SLC**	109.72 ± 8.18 ^H^	10,222.33 ± 807.93 ^BC^	1478.56 ± 75.68 ^FGH^	1097.82 ± 60.23 ^G^	21.57 ± 0.96 ^I^	23.51 ± 1.10 ^CD^	285.81 ± 17.38 ^CD^	131.06 ± 6.64 ^G^
	97.80–123.30	9218.00–11,422.00	1387.00–1570.00	984.60–1181.00	20.30–23.20	21.90–25.50	250.40–306.40	120.30–139.00
**SLU**	50.57 ± 6.32 ^FG^	13,794.22 ± 432.53 ^GH^	1856.11 ± 65.52 ^I^	1229.56 ± 43.17 ^H^	12.26 ± 0.27 ^DE^	27.64 ± 3.10 ^E^	453.46 ± 19.88 ^F^	37.93 ± 5.65 ^AB^
	43.20–61.10	13,204.00–14,571.00	1770.00–1990.00	1169.00–1290.00	11.80–12.60	24.40–32.60	431.20–487.20	31.00–47.50
**SLR**	29.97 ± 2.66 ^D^	11,289.67 ± 674.11 ^BCDEF^	1380.33 ± 120.13 ^EF^	997.88 ± 48.76 ^CDEFG^	10.91 ± 0.81 ^CD^	12.54 ± 0.79 ^A^	224.78 ± 13.30 ^AB^	28.97 ± 1.67 ^A^
	26.30–34.20	10,114.00–12,345.00	1234.00–1577.00	920.80–1090.00	10.10–12.30	11.20–13.70	210.20–247.20	27.20–32.40
**BCHI**	44.83 ± 5.08 ^EF^	10,576.78 ± 684.39 ^BCD^	1027.32 ± 94.02 ^C^	901.47 ± 56.72 ^ABCD^	16.56 ± 0.82 ^G^	31.08 ± 3.76 ^F^	525.49 ± 35.74 ^G^	80.86 ± 4.08 ^D^
	35.60–50.00	9218.00–11,471.00	916.30–1178.50	812.40–975.60	15.20–17.80	26.70–37.20	470.10–569.80	72.40–85.70
**CHY1**	14.47 ± 0.60 ^AB^	12,705.11 ± 1089.62 ^EFG^	1232.67 ± 163.91 ^DE^	905.68 ± 65.17 ^ABCD^	12.39 ± 0.44 ^DE^	31.19 ± 2.03 ^F^	846.29 ± 22.18 ^I^	61.27 ± 6.50 ^C^
	13.60–15.30	11,519.00–14,678.00	1055.00–1508.00	821.30–988.70	11.80–13.10	28.20–34.60	818.40–879.40	55.30–72.20
**CHY2**	54.00 ± 7.88 ^G^	11,693.56 ± 1165.32 ^CDEF^	1518.78 ± 76.23 ^FGH^	1040.59 ± 59.43 ^EFG^	10.11 ± 0.79 ^BC^	22.11 ± 1.56 ^BC^	465.11 ± 24.21 ^F^	109.81 ± 12.03 ^F^
	46.30–68.70	10,258.00–13,211.00	1391.00–1611.00	955.60–1118.00	9.17–11.30	20.40–24.50	420.80–492.20	89.40–120.40
**CHY3**	11.24 ± 0.76 ^A^	11,905.33 ± 1247.29 ^CDEF^	1115.00 ± 52.92 ^CD^	951.72 ± 80.38 ^BCDEF^	10.08 ± 0.41 ^BC^	21.73 ± 2.31 ^BC^	513.97 ± 33.17 ^G^	29.24 ± 1.80 ^A^
	10.20–12.20	10,122.00–13,318.00	1021.00–1181.00	840.60–1052.00	9.36–10.60	18.40–24.90	470.10–563.20	27.40–32.40
**CHF**	113.97 ± 7.53 ^H^	9635.33 ± 933.76 ^AB^	1588.00 ± 47.34 ^H^	990.81 ± 82.01 ^CDEFG^	13.67 ± 1.32 ^EF^	22.98 ± 1.64 ^BCD^	711.44 ± 25.45 ^H^	108.82 ± 6.13 ^F^
	102.00–124.40	8325.00–10,812.00	1511.00–1645.00	871.50–1071.00	11.40–14.80	20.50–25.10	672.40–755.20	101.40–120.50
**IA1**	19.22 ± 2.21 ^B^	12,805.33 ± 951.79 ^FG^	1602.67 ± 41.79 ^H^	1233.44 ± 68.09 ^H^	19.48 ± 1.94 ^H^	23.61 ± 1.29 ^CD^	202.77 ± 12.48 ^A^	33.56 ± 2.48 ^AB^
	15.40–21.60	11,401.00–13,934.00	1540.00–1656.00	1133.00–1335.00	17.00–23.20	21.80–25.50	185.40–220.10	28.80–36.50
**IA2**	17.87 ± 1.52 ^AB^	12,146.67 ± 1447.65 ^DEFG^	1465.00 ± 119.20 ^FGH^	1035.662 ± 80.96 ^EFG^	15.34 ± 0.62 ^FG^	35.03 ± 2.28 ^G^	434.61 ± 35.96 ^F^	42.42 ± 4.24 ^B^
	16.20–21.30	9762.00–13,920.00	1229.00–1588.00	909.50–1155.00	14.50–16.30	31.90–38.10	380.40–472.50	36.10–49.10
**ID**	49.00 ± 3.31 ^FG^	11,498.67 ± 701.82 ^CDEF^	1482.67 ± 97.36 ^FGH^	961.62 ± 26.55 ^BCDEF^	14.88 ± 1.41 ^FG^	27.48 ± 1.81 ^E^	250.64 ± 15.26 ^BC^	34.32 ± 3.79 ^AB^
	44.20–54.40	10,218.00–12,545.00	1325.00–1591.00	920.30–991.90	12.90–16.90	24.30–29.10	228.10–272.50	28.80–38.20
**Total**	**42.73 ± 27.42**	**11,649.88 ± 1794.61**	**1272.95 ± 347.65**	**998.25 ± 127.71**	**13.87 ± 4.73**	**25.12 ± 5.44**	**447.73 ± 193.97**	**70.79 ± 41.90**
	**10.20–124.40**	**7719.00–17,878.00**	**411.40–1990.00**	**705.40–1335.00**	**5.15–26.20**	**11.20–34.00**	**185.40–879.40**	**22.30–194.60**

**Table 2:** The distribution of the attribute values of nodes in the tree with the selected teas.

Type of Tea	Classification Tree Structure [mg/kg]
Ir	Mn ≤ 766.25; Cu > 16.05; Mn > 621.1
CHY1	Zn > 26.65; Cu ≤ 16.05; Mn > 621.1
SLC	Fe > 79.25; Cu > 12.65; Ca > 1211.5; Mn ≤ 330.4 and ≤ 621.1
SLR	Zn ≤ 17.1; Cu ≤ 12.26; Ca > 1211.5; Mn ≤ 330.4 and ≤ 621.1

**Table 3:** Characteristic of the investigated black teas.

Voucher Specimen Number	Country	Region/Type	Origin	Form
JO	Japan	Organic	original	loose
JS	Japan	Shizuoka Prefecture	original	loose
NI1	Nepal	Ilam	original	loose
NI2	Nepal	Ilam	original	loose
NI3	Nepal	Ilam	original	loose
NI4	Nepal	Ilam	original	loose
K1	Kenya	Marinyn	original	loose
K2	Kenya	Mount Kenya	original	loose
Ir	Iran	Lahijan	original	loose
SLC	Sri Lanka	Central Province	original	loose
SLU	Sri Lanka	Uva	original	loose
SLR	Sri Lanka	Ruhuna	original	loose
BCHI	China/India	Not provided	blended	loose
CHY1	China	Yunnan	original	loose
CHY2	China	Yunnan	original	loose
CHY3	China	Yunnan	original	loose
CHF	China	Fuijan	original	loose
IA1	India	Assam	original	loose
IA2	India	Assam	original	loose
ID	India	Darjeeling	original	loose

## Supplementary Materials

The following are available online. Figure S1: Classification tree for samples of tea (full version).
