# Supplementary material for: Analytical Assessment of Bioelements in Various Types of Black Teas from Different Geographical Origins in View of Chemometric Approach
Source: Molecules. 2021 Oct 4;26(19):6017. doi: 10.3390/molecules26196017 (PMC8512582; doi:10.3390/molecules26196017)
Supplement: Supplementary file 1 [file molecules-26-06017-s001.zip › molecules-1399419-supplementary.pdf]

Supplementary file.

Analytical assessment of bioelements in various types of black teas from different geographical origins in view of chemometric approach

Wojciech Koch [1]*, Wirginia Kukula-Koch [2], Marcin Czop [3], Tomasz Baj [2], Janusz Kocki [3], Piotr Bawiec [1], Roser Olives Casasnovas [1,4], Anna Głowniak-Lipa [5] and Kazimierz Głowniak [5]

[1] Chair and Department of Food and Nutrition, Medical University of Lublin, 4a Chodźki Str., 20-093 Lublin, Poland Piotr.bawiec@wp.pl (P.B.)
[2] Chair and Department of Pharmacognosy, Medical University of Lublin, 1 Chodźki Str., 20-093 Lublin, Poland; virginia.kukula@gmail.com (W.K.-K.)
[3] Department of Clinical Genetics, Medical University of Lublin, 11 Radziwiłłowska Str., 20-093 Lublin, Poland; marcin.czop@umlub.pl (M.C.); janusz.kocki@umlub.pl (J.K.)
[4] University of Barcelona, Spain; roserolives@hotmail.com
[5] Department of Cosmetology, University of Information Technology and Management in Rzeszów, Kielnarowa 386a, 36-020 Tyczyn, Poland; aglowniak@wsiz.edu.pl (A.G.L.); kglowniak@pharmacognosy.org (K.G.)
* Correspondence: kochw@interia.pl; Tel.: +48-81-448-7143

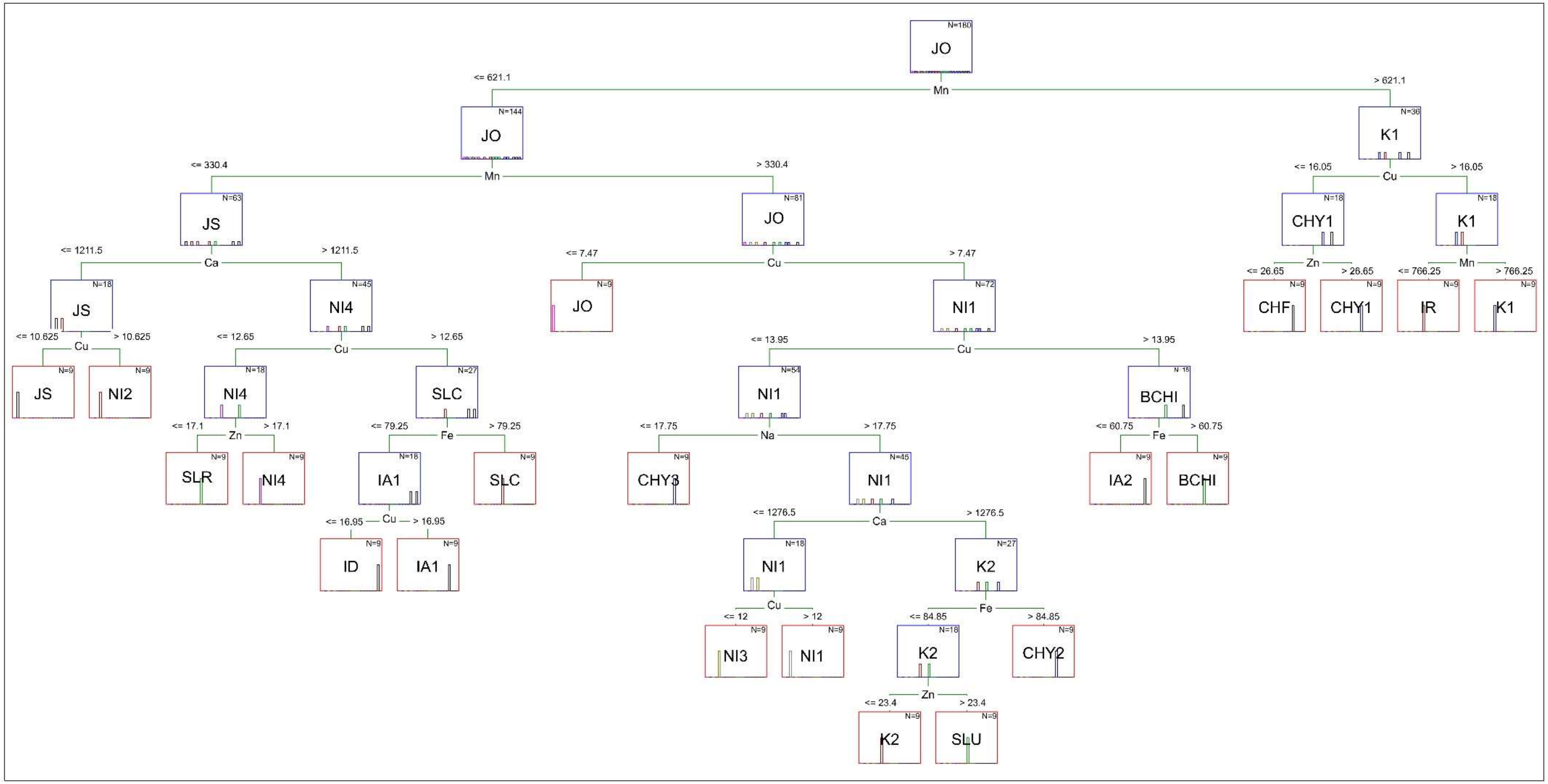

Figure S1. Classification tree for samples of tea (full version).
